# Bent or broken: analysis of set screw fracture in the TFNa implant

**DOI:** 10.1186/s10195-021-00594-8

**Published:** 2021-08-03

**Authors:** Matthew Klima

**Affiliations:** Sarasota Memorial Hospital, 1921 Waldemere Street, Suite 504, Sarasota, FL 34239 USA

**Keywords:** Implant failure, Cephalomedullary nails, Locking mechanism failure, TFNa, Set screw, Set screw fracture

## Abstract

**Objectives:**

To evaluate set screw fracture in the Trochanteric Femoral Nail Advanced implant (TFNa, Synthes, West Chester, PA) and to identify additional mechanisms of set screw failure in the TFNa.

**Materials and methods:**

Patients who had experienced failure after open reduction and internal fixation (ORIF) with the TFNa were identified. TFNa implants were explanted and inspected following revision surgery. Medical device reports (MDRs) and manufacturer’s inspection reports describing similar failures for the TFNa in the United States Food and Drug Administration (FDA) Manufacturer and User Facility Device Experience (MAUDE) database were also reviewed.

**Results:**

Four set screw fractures that occurred at a level II trauma center were observed. Sixty-seven reported failures were identified in the MAUDE database for review. Twenty-eight failed implants were returned to the manufacturer for inspection with a published inspection report available for analysis. Set screw fractures can occur in the TFNa when the set screw is deployed prematurely into the proximal screw aperture prior to blade/screw insertion. The set screw can also bend and deform if it is advanced against a helical blade/lag screw that is not fully seated into position, thereby potentially compromising its function.

**Conclusion:**

The TFNa set screw allows for potential fracture during implant insertion leading to uncontrolled collapse, early excessive proximal femoral shortening, and rotational instability of the helical blade/lag screw. Similar failures in the TFNa can be prevented by having the surgeon inspect the proximal screw aperture after attachment of the proximal aiming aim to ensure the set screw has not been deployed prematurely.

**Level of evidence:**

Therapeutic Level III.

**Supplementary Information:**

The online version contains supplementary material available at 10.1186/s10195-021-00594-8.

## Introduction

Mechanical failure after fixation of proximal femur fractures with cephalomedullary nails has been well described in terms of implant “cut out” from the femoral head and implant fracture [1–16]. Fewer studies have described the proximal locking mechanism failure because of its rare occurrence, and limited information exists to demonstrate the clinical presentation of those failures. Since sliding and rotation of the lag screw/helical blade are controlled in most cephalomedullary nails by a preloaded set screw, failure of that set screw could result in uncontrolled collapse, non-union, shortening, medial migration, and loss of reduction [13, 17].

This study aimed to evaluate set screw/locking mechanism fracture in the TFNa implant to answer the following questions; What causes the implant to fracture, and how can failures be identified? Are there additional mechanisms by which set screw failure can occur? Due to the paucity of information available published on this topic, the FDA’s MAUDE database was employed for reports of similar failures in the TFNa that could assist the investigation into the mechanism. The FDA’s MAUDE database has previously been used to analyze implant failures provided that the data are not used to compare rates of failures between implants within the same category [16, 18, 19]. For purposes of this study, the manufacturer’s inspection reports available in the database of failed implants could provide useful insight into the circumstances and causes of the failure as determined by the manufacturer.

## Clinical presentation of a fractured set screw: sample case report

**Fig. 1:**
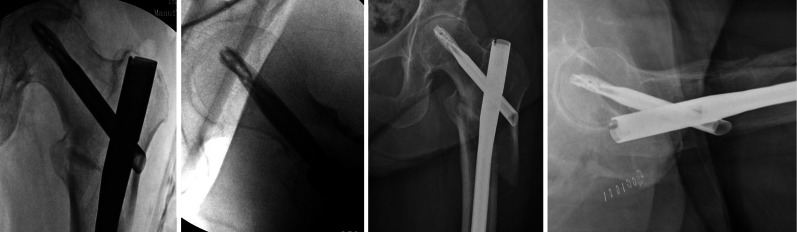
Pictured left are the AP and lateral views of the hip obtained following the index procedure. Pictured right are the repeat radiographs taken 2 weeks postoperatively that show interval displacement of the fracture, loss of reduction, and shortening of the proximal femur

An 86-year-old female sustained an unstable reverse obliquity intertrochanteric femur fracture (AO A3) following a ground-level fall. The patient was an independent ambulator prior to initial injury and was independent with functional tasks, ASA category 3. Operative treatment was performed by the on-call orthopedic surgeon the following day within 24 h of the injury. Fixation was achieved with a TFNa implant, 12 mm × 420 mm × 130°. Intraoperative fluoroscopic images from the procedure are included as part of Fig. 1. Two distal interlocking screws were placed, and static locking of proximal set screw was performed by the operating surgeon. Operative time was approximately 60 min. The patient’s pain level was 5 out of 10 consistently in the early postoperative period. The patient ambulated weight bearing as tolerated for a distance of 20 ft with a walker and minimal assistance. Postoperative hospitalization was uncomplicated, and the patient was discharged to a rehabilitation facility on postoperative day 4.

**Fig. 2:**
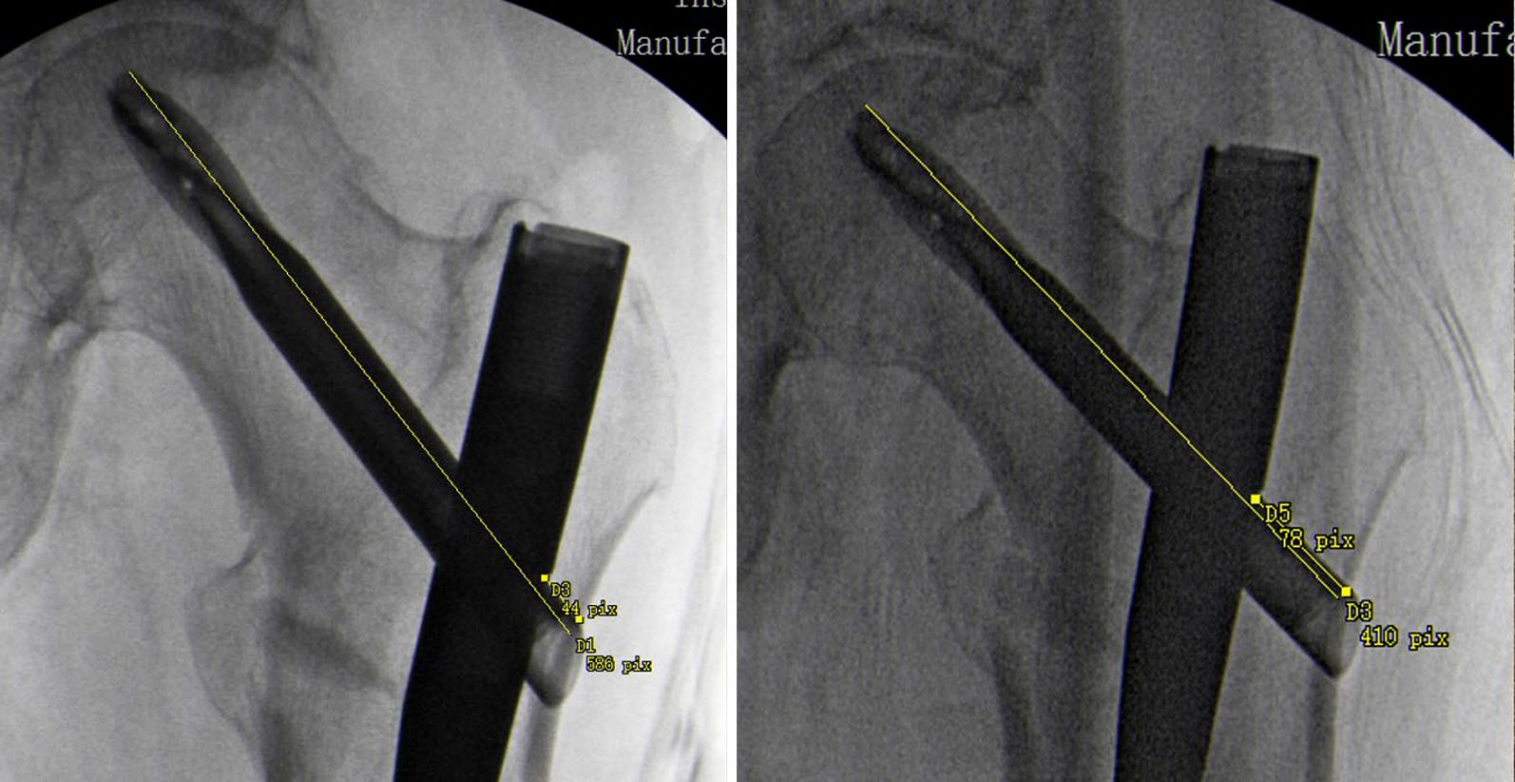
Depicted is the technique for measuring proximal femoral shortening as described by Serrano et al. The measurements taken from the drawn lines are compared with the length of the helical blade. The proximal femur has shortened 1.2 cm from the images obtained during the initial procedure (left) to images obtained 12 days later (right)

While at a rehabilitation facility, the patient noted progressive pain level increases, wherein the pain levels spiked to 9 out of 10, particularly with transfers. Increasing difficulty with ambulation and functional mobility were noted, and the patient deteriorated to ambulating less than 5 ft with a walker and moderate assistance. Eventually, complete ambulation became impossible because of pain, and repeat imaging was ordered on postoperative day 12. The repeat radiographs revealed interval displacement of the fracture with loss of reduction and are included as part of Fig. 1. Due to loss of reduction and progressive functional decline, the operating surgeon consulted the local orthopedic trauma service. Upon examination, the patient had severe pain with internal and external rotation of the hip and inability to perform a straight leg raise. Interval X-rays were obtained showing proximal femoral shortening of 1.2 cm as measured by Serrano (Fig. 2) [20].

**Fig. 3:**
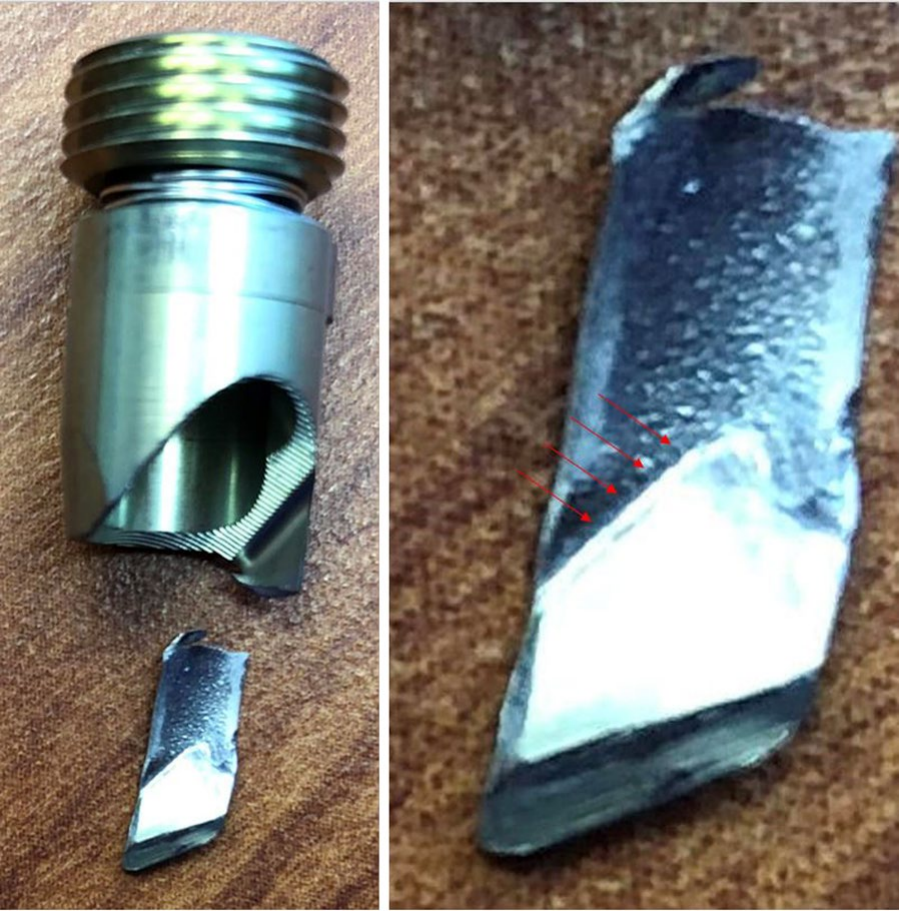
A fractured set screw for the TFNa was discovered after removal and disassembly of the implant. Arrows point to engaging defect of the long prong with corresponding lesions on the helical blade consistent with fracture of the prong by the edge of the blade

Urgent revision surgery was performed the following day. The implant was revised to a rotationally stable construct with dual integrated screws, Intertan (Smith and Nephew, Memphis, TN). The failed TFNa implant was fully disassembled and inspected on the back table. The set screw/proximal locking prong was found to be broken at its base (Fig. 3). Following explant of the failed hardware, anatomical reduction of the fracture was achieved with a Verbrugge clamp and cable fixation. Total operative time was 1 h 56 min with 200 cc blood loss.

**Fig. 4:**
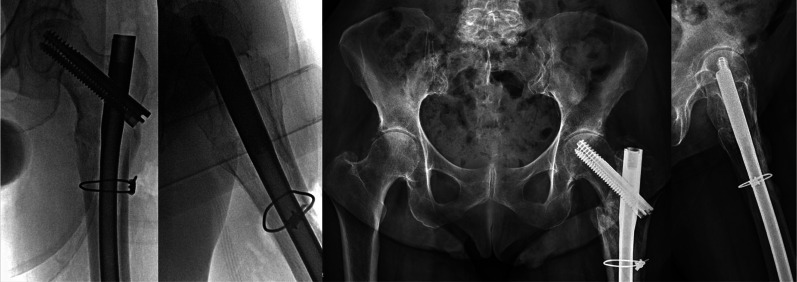
(Left) Anteroposterior (AP) and lateral view of the left hip obtained at the time of revision surgery showing restoration of proximal femoral length and neck shaft angle. (Right) AP pelvis and lateral hip X-ray obtained 6 months postoperatively showing full union of the fracture with no interval shortening

After the revision procedure, the patient immediately noted significant pain level improvement from 8–9 to 2–3. Ambulatory distance increased to 50 ft in the early postoperative period, and the patient returned to rehabilitation on postoperative day 3. Continued improvements in functional status were noted as the patient was able to ambulate independently over 150 ft with a walker upon discharge from rehabilitation. The patient eventually returned to baseline functional status with no complaints of hip pain. The X-rays obtained at 6 months after the revision procedure show full union, maintenance of reduction, and no appreciable proximal femoral shortening (Fig. 4).

## Materials and methods

Institutional Review Board approval was obtained to perform this study, and patient consent was obtained for publication. Patients that experienced failure, defined as a need for a secondary procedure, after ORIF of a proximal femur fracture with a cephalomedullary nail were identified from 2018 to 2020 at a level II trauma center. Implants were removed and inspected following revision surgery. TFNa implants were fully disassembled after sterilization to allow for thorough inspection of the set screw. Retrospective review of the patient’s medical record was performed to determine if the medical device failure may have impacted outcome. A medical device report was submitted directly to the FDA by the operating surgeon in the event that a set screw fracture was thought to have contributed to a negative patient outcome.

Individual medical device reports made to the FDA from January 2015 to October 2019 were reviewed along with available manufacturer inspection reports of failed TFNa implants.

Duplicate reports included in the MAUDE database search were removed prior to analysis. Individual medical device reports were reviewed for a description of the clinical presentation of the failures. Individual inspection medical device reports from the manufacturer of failed implants were reviewed to identify the mechanism of failure, and if the mechanism was consistent with the description of the clinical presentation.

## Results

Twenty-seven cephalomedullary nails were explanted, sterilized, and available for inspection, seven of which were TFNa implants. Four fractured set screws were observed. One was fractured at the time of implantation, resulting in early failure as described in the sample case report. One fracture was discovered in a case of medial migration of the proximal lag screw, one fracture was associated with a proximal femur non-union, and one fracture occurred during removal of the helical blade at the time of revision ORIF for non-union.

A total of 2767 MDRs were reviewed pertaining to “break” events in the TFNa, Gamma 3 (Stryker, Mahwah, NJ), and Intertan implants. Among those three implants, only the TFNa set screw was reported to have fractured. Altogether, 117 reports of locking mechanism failure in the TFNa were obtained from a search of “break” events of the MAUDE database. Sixty-seven reports were available for review after eliminating duplicate reports. A summary of the categorical data is included in Table 1. Twenty-eight of those failed TFNa implants were returned to the manufacturer for inspection with a published inspection report.

**Table 1:** Reported failures of the set/screw locking mechanism of TFNa in the FDA’s MAUDE database from 2015 to 2019, and the mechanism of failure in the observed cases of set screw fracture

	Reported failures
Total reported set screw failures in the MAUDE database	67
Reported failures with helical blade insertion	48
Reported failures with lag screw insertion	19
Failure to lock helical blade/lag screw after fully deployed	13
Lock prong obstructed helical blade/lag screw insertion	11
Fractured lock prong remained as retained foreign body	3
Damage to locking mechanism associated with non-union	9
Uncontrolled collapse and early loss of reduction	8
Medial migration of the helical blade/lag screw	8
Damage to locking mechanism associated with cut out	4
Total observed cases of fractured lock prong	4
Medial migration of lag screw	1
Early loss of reduction, uncontrolled collapse	1
Fractured during removal resulting in retained foreign body	1
Non-union	1

## Discussion

**Fig. 5:**
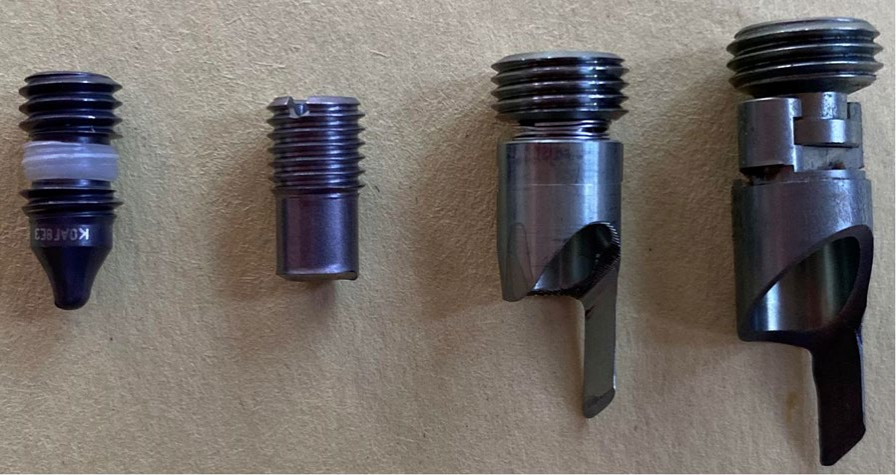
Pictured from left to right are the set screws for the Gamma 3, Intertan, TFNa, and TFN cephalomedullary nails. The set screws for the TFNa and TFN are unique as they have two separate pieces. The components are divided into the upper portion, or lock drive, and the lower portion, or lock prong. The lock prong in the TFNa appears smaller and thinner in size than its predecessor, the TFN. A spring has also been added in the TFNa

Few studies have described set screw/proximal locking mechanism failure because of its rare occurrence, and limited information exists to demonstrate the clinical presentation of those failures [17, 20, 21]. While sliding and rotation of the lag screw/helical blade are controlled in most cephalomedullary nails by a preloaded set screw, the design of the set screw in the TFNa is novel compared with other CMN (Fig. 5). As opposed to the other implants pictured, the design of the TFNa set screw allows for it to potentially fracture resulting in several different scenarios of failure.

One such failure, described in detail in the sample case report, illustrates the impact of TFNa set screw fracture on excessive shortening, or uncontrolled collapse. Although shortening is to be anticipated via a mechanism of controlled collapse by which most intertrochanteric femur fractures achieve union, the extent to which proximal femoral shortening is predictive of construct failure and “uncontrolled” collapse has yet to be fully determined [22]. “Controlled” collapse, or expected collapse, has been quantified by previous studies even in the most unstable intertrochanteric fractures to be on average 5 mm at 3 months with single screw fixation [23, 24]. Even in cohorts with set screws unlocked a quarter turn to facilitate some sliding, shortening remained on average 5 mm to 1 cm at 3 months [24]. With shortening of 1.2 cm, the patient would have experienced altered gait mechanics and a poor functional outcome, demonstrated by previous studies with shortening of 8 mm and 5 mm, respectively [9, 25–27]. Defining “uncontrolled” collapse as a clinical diagnosis made of a spectrum of symptoms to include unexpected proximal femoral shortening combined with significant pain, and change in ambulatory status may provide a useful barometer by which the need for revision surgical intervention can be demonstrated acutely. Earlier intervention is important in this particular patient population because of the increased risks associated with prolonged decline in functional status [28, 29].

**Fig. 6:**
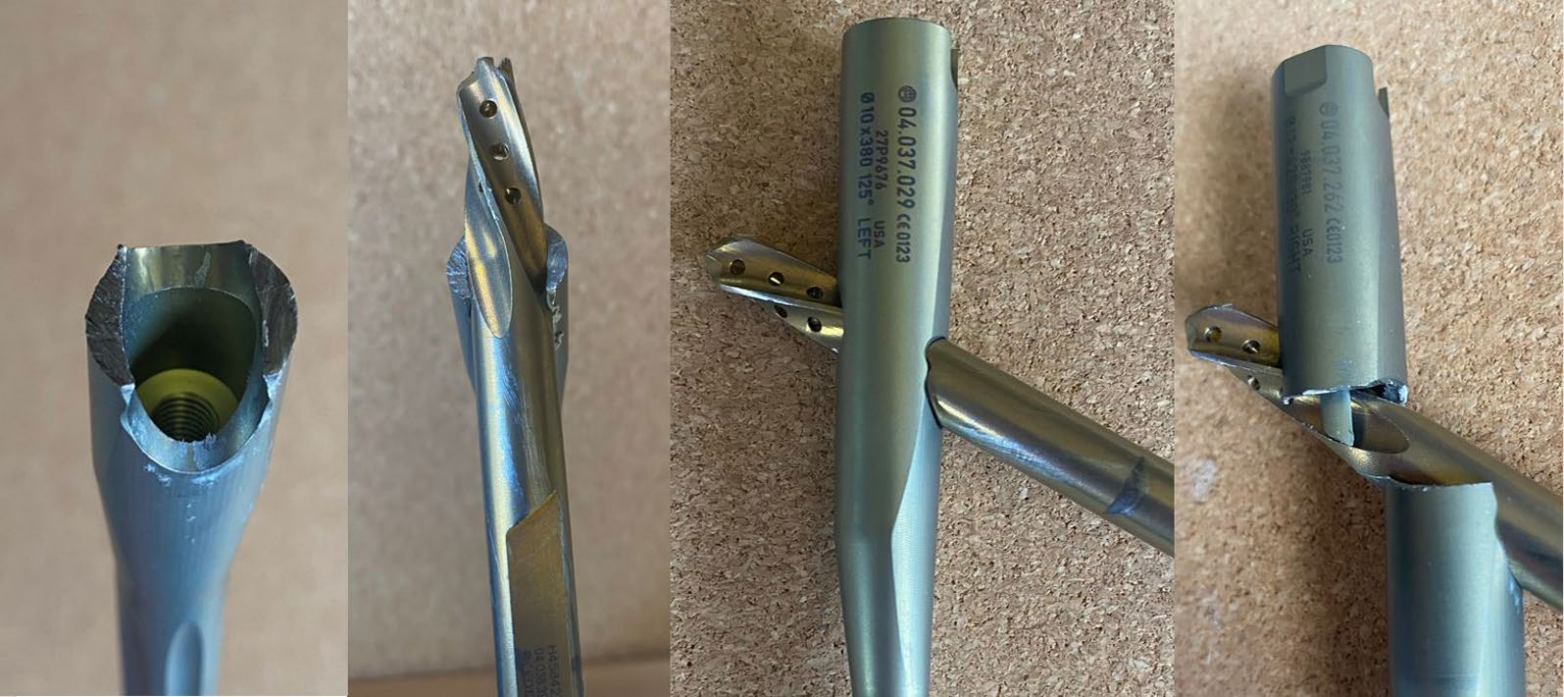
Pictured from left to right: (1) Looking down at the lateral aspect of the lower half of a sectioned TFNa implant thru the proximal screw aperture. (2) The diameter of this sectioned proximal screw aperture is completely filled with insertion of the helical blade. (3) When the set screw is prematurely deployed, it acts as a mechanical block to the advancing helical blade. The blade is unable to advance any further than pictured without fracture of the locking prong. (4) Sectioned TFNa implant that has been displaced to show the direct contact of the helical blade against the locking prong when it has been deployed prior to blade/screw insertion

**Fig. 7:**
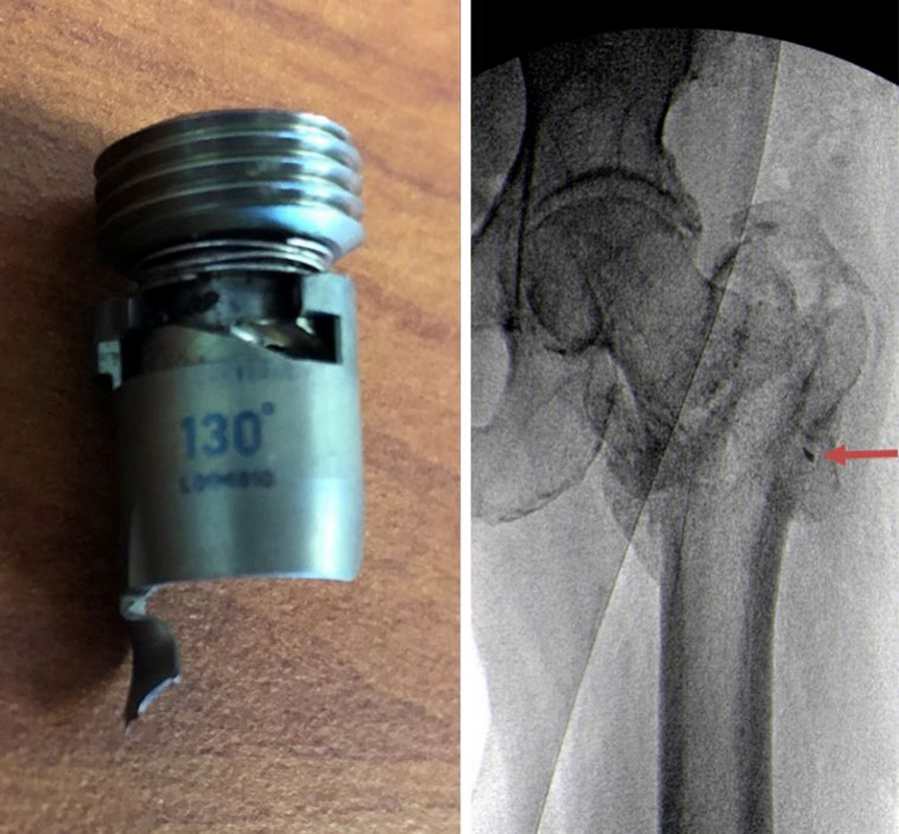
Visualized on the left is the characteristic deformity and fracture of the TFNa set screw that occurs if the lock prong is damaged during implant extraction. Failure to fully elevate the set screw prior to helical blade removal will cause the lock prong to deform and fracture more distally toward its tip. The fractured lock prong in this case remained within the patient as a retained foreign body visible on X-ray marked by the arrow in the image to the right

The locking mechanism for the TFNa is composed of two major components coupled by a spring, an upper portion or “lock drive” and a lower portion or “lock prong.” Per the manufacturer, the reported fractures occur when the lock prong is advanced into the proximal screw aperture prior to blade/screw insertion (Fig. 6). Either the blade/screw or the stepped reamer can strike the lock prong as they pass through the proximal aperture, resulting in prong fracture at its base. This occurs when the wrong screwdriver is mistakenly used to attach the aiming arm to the implant, thereby advancing the lock prong into the bore of the proximal screw aperture. By design, the screw driver to advance to set screw can still pass through the various pieces of the aiming arm assembly and engage the locking mechanism. Additionally, breakage of the lock prong can occur during implant extraction when the lock prong has not been fully retracted prior to blade/lag screw removal. Under these circumstances, breakage of the lock prong would not be associated with an adverse event unless the broken piece escaped the surgical field and remained in the patient as a retained foreign body (Fig. 7). To prevent locking mechanism fractures when using the TFNa implant, we recommend the surgeon inspect the proximal screw aperture after attachment of the proximal aiming arm prior to insertion of the implant to ensure the locking prong has not been deployed.

Altogether, eight reported failures in the MAUDE database were related to medial migration of the blade/screw in the TFNa, although none of the manufacturer inspection reports attributed this complication to damage of the locking mechanism. For the TFNa, there is an edge along the lateral surface of the helical blade/lag screw that engages the lock prong, limiting how far the blade/screw can migrate medially or laterally. In one of the observed failures, medial migration of the lag screw was preceded by severe collapse that progressed up until the lock prong prevented further slide of the lag screw in the proximal aperture. Predictably, the lock prong eventually fractured, which ultimately permitted escape of the lag screw from the proximal implant as it then migrated medially into the abdomen.

**Fig. 8:**
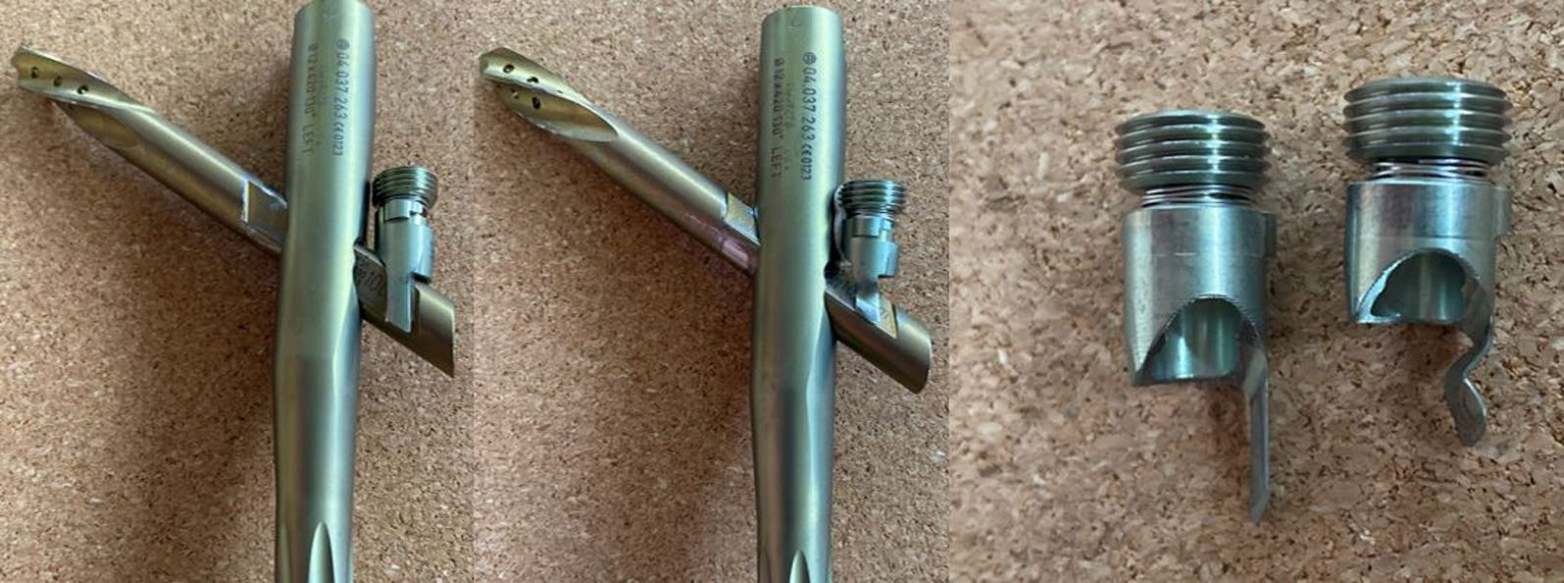
Far left, the helical blade has been inserted correctly so that the orientation of the recess on the side of the helical blade docks with the locking prong correctly and allows the set screw to be seated fully. Pictured middle, with an under-inserted helical blade, this recess is no longer aligned with the trajectory of the set screw. In this scenario, the set screw will advance until the lock prong contacts the superior aspect of the blade/screw resulting in deformation. Pictured right, a comparison of an intact locking prong and a deformed locking prong

In addition to implant breakage, the locking prong can also bend or deform if it is advanced against a helical blade/lag screw that is not fully seated into position. The aiming arm of the TFNa is designed so that, when the blade is fully seated, the recess on the lateral side of the blade/screw will be oriented to allow passage of the advancing lock prong (Fig. 8). If the operating surgeon attempted to insert a helical blade that was too long and stopped short of being fully inserted, the recess on the helical blade would not be oriented correctly to allow for passage of the locking prong into position. In this scenario, the tip of the locking prong would engage the blade/screw along its superior aspect, and deform even when inserted with a torque limiting screw driver. A deformed locking prong would compromise rotational control of the helical blade/lag screw and the ability to resist shortening. Backing off the set screw in these circumstances, which is recommended by the manufacturer to allow for blade/screw sliding, would then result in a completely unlocked proximal construct with lack of rotational control.

Although some set screw failures/fractures can be attributed to user technique during insertion, design differences in the set screw themselves may also contribute to additional sources of failure (Fig. 5). Key differences in the newer design include the smaller size of the set screw components in the TFNa compared with the first-generation TFN, and the addition of a coupling spring. During deployment, the lock drive is engaged by a screwdriver and begins to rotate within the threads of the proximal implant while advancing the lower lock prong. While the lock drive is intended to rotate to function correctly, the lock prong is not, and any rotational forces that are translated to the lock prong while it is advanced could result in jamming/bending of the lock prong against the implant wall (Additional file 1: Video S1). The operating surgeon would note increased resistance or difficulty advancing the set screw while attempting to lock the proximal implant. Four similar reports were noted among the MDRs in the MAUDE database. Hence, with a smaller lock prong, there is less resistance to any accessory rotation translated by the lock drive, increasing the possibility for jamming/bending into the implant wall. Inappropriate tensioning of the coupling spring could also force the lock prong to rotate more while the locking mechanism is advanced, causing jamming/bending. There have been additional studies by the manufacturer to evaluate for possible changes in set screw position during transit that would result in the lock prong arriving in the deployed position prior to opening. Accordingly, none of these studies was able to re-create such a change due to transit. There have been two FDA recalls for TFNa pertaining to a set screw that was positioned too high in the proximal implant, preventing attachment of the aiming arm [30]. However, no recall has ever been issued to date for a set screw positioned too low in the proximal nail.

Since the set screw is internal to the proximal aspect of the implant, intraoperative damage and compromise to the locking mechanism may potentially go unrecognized. Among the reported failures in the MAUDE database, premature deployment of the locking prong into the aperture was discovered when the helical blade was unable to pass through the proximal screw aperture owing to increased resistance or blockage. Concerning were reports in which the helical blade became incarcerated within the proximal screw aperture and was unable to be disimpacted from the cephalomedullary nail in the operative theater. The inspection report from the manufacturer pertaining to this event describes it as a “cold welding” that required the implant pieces be separated in their lab after their return en block. Per the technique guide, failures can also be detected by checking to see if blade/screw is able to be rotated within the proximal screw aperture via the insertion handle following locking.

As the locking mechanism is internal to the implant and the lock prong is poorly visualized on fluoroscopy, failures may be difficult to detect. As per the author’s experience, use of CT scan to assist in detecting failure of the TFNa locking mechanism is limited because of the actual size of the locking prong. Artifact in the area of the locking mechanism is typically present on CT, preventing accurate imaging of deformed and broken pieces. CT scan can be successful in measuring the height of the lock drive in relation to the top of the nail, and when fully deployed, it averages 2.2 cm from the top of the implant. In cases of under-insertion of the blade/screw with deformity of the locking prong or in cases where the locking mechanism was not fully advanced, it would result in a heightened position of the lock drive, as discovered in a non-union case at our institution.

There are several limitations to our study. As there is little information available on this important topic, fully understanding the multitudes of factors contributing to the mechanism of failure is challenging. The MAUDE database can be a valuable resource in these instances as it provides a large collection of reports pertaining to medical device failures in the USA.

The benefits of this database are offset by several important limitations. Key information can often be missing in the reports, particularly when that information has not been deemed relevant to the device failure that affects both the quality and validity of the data [16].

Protected patient information can be missing from the reports, which may limit the scope of the analysis that can accurately be performed [16, 18]. Each report must be carefully reviewed and filtered to allow only the best-quality information to be considered, as we did in this study. Often the information in an MDR pertaining to a failure can reflect the opinions of staff or sales representatives rather than an evaluation from a qualified engineer. In this study, the MDRs did not provide much substance, and our review of the MAUDE data focused on the inspection reports from the manufacturer. These inspection reports provide a detailed examination of the implant by when it is returned to the manufacturer. These reports confirm or refute the complaint against the device, and provide a detailed explanation of the mechanism of failure that only increases the validity of our conclusions. With the MAUDE database, it is impossible to determine the rate of failure as the total number of devices implanted is unknown, and at no time does this study attempt to compare or establish rates of failure.

Therefore, as opposed to other implants, the TFNa set screw allows for potential fracture during implant insertion leading to uncontrolled collapse, excessive proximal femoral shortening, and construct failure. Per the manufacturer, these reported failures occur when the lock prong is advanced into the proximal screw aperture prior to blade/screw insertion. In addition to breakage, we discovered several other scenarios where the lock prong can deform, compromising both control of rotation and shortening of the helical blade/lag screw. We recommend the surgeon inspect the proximal screw aperture of the TFNa implant after attachment of the proximal aiming arm to ensure that the locking prong has not been deployed prior to insertion. Since the set screw is internal to the proximal aspect of the nail, and proper inspection requires complete disassembly, many locking mechanism failures potentially are unrecognized and underreported.

## Supplementary Information

**Additional file 1: Video S1.** Depicted is the lock prong of the TFNa set screw rotating in the proximal implant as it is advanced. The dual component set screw of the TFNa is designed so that only the upper portion, or lock drive, should rotate as it is deployed. Accessory rotation of the lock prong outside of its intended path as it is advanced could result in jamming/bending of the lock prong against the implant wall prior to it being fully seated in its correct position next to the helical blade.

## Data Availability

Not applicable.
